# Identification of nuclear genes affecting 2-Deoxyglucose resistance in *Schizosaccharomyces pombe*

**DOI:** 10.1093/femsyr/fow061

**Published:** 2016-07-31

**Authors:** Akshay Vishwanatha, Charalampos Rallis, Shubha Bevkal Subramanyaswamy, Cletus Joseph Michael D'Souza, Jürg Bähler, Martin Ernst Schweingruber

**Affiliations:** 1 Department of Studies in Biochemistry, University of Mysore, Manasagangotri, Mysuru 570 006, Karnataka, India; 2 Research Department of Genetics, Evolution and Environment, UCL Institute of Healthy Ageing, University College London, London WC1E 6BT, UK; 3 Institute of Cell Biology, University of Bern, Baltzerstrasse 4, CH-3012 Bern, Switzerland

**Keywords:** fission yeast, glucose metabolism, 2-Deoxyglucose resistance, invertase activity, *odr1* overexpression, deletion library

## Abstract

2-Deoxyglucose (2-DG) is a toxic glucose analog. To identify genes involved in 2-DG toxicity in *Schizosaccharomyces pombe*, we screened a wild-type overexpression library for genes which render cells 2-DG resistant. A gene we termed *odr1*, encoding an uncharacterized hydrolase, led to strong resistance and altered invertase expression when overexpressed. We speculate that Odr1 neutralizes the toxic form of 2-DG, similar to the *Saccharomyces cerevisiae* Dog1 and Dog2 phosphatases which dephosphorylate 2-DG-6-phosphate synthesized by hexokinase. In a complementary approach, we screened a haploid deletion library to identify 2-DG-resistant mutants. This screen identified the genes *snf5, ypa1, pas1* and *pho7*. In liquid medium, deletions of these genes conferred 2-DG resistance preferentially under glucose-repressed conditions. The deletion mutants expressed invertase activity more constitutively than the control strain, indicating defects in the control of glucose repression. No *S. cerevisiae* orthologs of the *pho7* gene is known, and no 2-DG resistance has been reported for any of the deletion mutants of the other genes identified here. Moreover, 2-DG leads to derepressed invertase activity in *S. pombe*, while in *S. cerevisiae* it becomes repressed. Taken together, these findings suggest that mechanisms involved in 2-DG resistance differ between budding and fission yeasts.

## Teaser

Genes that are required to provide resistance to the anticancer molecule 2-Deoxyglucose when overexpressed or deleted in fission yeast have been identified.

## INTRODUCTION

There are two main reasons to study the mechanisms of action of the toxic glucose analog 2-Deoxyglucose (2-DG). First, in the budding yeast *Saccharomyces cerevisiae* 2-DG inhibits growth, affects cell wall synthesis, causes abnormal cell morphology and cell lysis, and interferes with glucose metabolism (Biely *et al.*^1971^; Krátký, Biely and Bauer 1975). A series of pioneering papers (Zimmermann and Scheel 1977; Entian and Zimmermann 1980; Neigeborn and Carlson 1987) have identified mutants resistant to 2-DG, and corresponding genes have been characterized leading to insights into the underlying mechanisms (for additional references, see McCartney *et al.*^2014^). Second, 2-DG shows anticancer activity in humans by inhibiting tumor growth with yeast providing a model system to understand its mode of action (Cairns, Harris and Mak 2011; Raez *et al.*^2013^). Aerobic glycolysis is a metabolic pathway that is of particular importance to cancer cells for generating energy, and 2-DG is thought to impede this pathway by inhibiting several of its enzymes (Pelicano *et al.*^2006^). Glycolysis is also the metabolic pathway used by rapidly proliferating yeast cells to ferment glucose to ethanol. After cellular uptake, hexokinase phosphorylates 2-DG to the highly toxic 2-DG-6-phosphate, which in turn cannot be further converted to fructose-6-phosphate (Jaspers and van Steveninck 1975; Lobo and Maitra 1977).

The fission yeast, *Schizosaccharomyces pombe*, is only remotely related to budding yeast and provides a valuable complementary model organism, being in several ways more closely related to humans than to budding yeast (Hoffman, Wood and Fantes 2015). Research by the Hoffman laboratory and others on glucose metabolism has advanced our understanding of glucose signaling in fission yeast. Glucose is sensed by the Git/Protein Kinase A (PKA) pathway (Hoffman 2005) and the glucose repression pathway involving glucose-6-phosphate (Roux *et al.*^2009^). Based on work in *S. cerevisiae*, 2-DG alters glucose sensing and induces a glucose starvation signal (O'Donnell *et al.*^2015^). During glucose starvation, *S. pombe* PKA forms a complex with its regulatory subunit, and it becomes phosphorylated and re-localized to the cytoplasm (Gupta *et al.*^2011^). Glucose starvation triggers the stress-activated protein kinase pathway in *S. pombe* (Madrid *et al.*^2006^, ^2013^), resulting in a gene expression response (Madrid *et al.*^2004^; Kato, Kira and Kawamukai 2013).

In *S. pombe*, 2-DG leads to deformed cells that lyse at the site of glucan synthesis (Megnet 1965; Johnson 1968). In addition, the 2-DG-resistant *std1* mutant shows defects in glucose transport (Mehta *et al.*^1998^), and other 2-DG-resistant mutants exhibiting derepressed invertase activity and impaired glucose uptake map to four different genetic loci (Kig, Turkel and Temizkan 2005). Some glucose transport-deficient *S. pombe* mutants are also resistant to 2-DG, but 2-DG resistance and glucose transport deficiency have been attributed to different genetic loci (Milbradt and Höfer 1994). Here, we systematically identify genes that cause 2-DG resistance when overexpressed or when deleted to obtain functional information on 2-DG action in fission yeast.

## MATERIALS AND METHODS

### Media, reagents and strains

Standard methods and media were used for growth of *Schizosaccharomyces pombe* strains. The minimal media used were Edinburgh minimal medium 2 (EMM2) (Moreno, Klar and Nurse 1991) and MM as described (Schweingruber and Edenharter 1990), containing either 2% or 0.5% glucose. In contrast to EMM, MM allows to study growth as well as mating and sporulation. It was therefore chosen to characterize the 2-DG-resistant strains. The two media differ in their ammonium content. All media contained leucine, adenine and uracil supplements as required by the strains at 50 μg/ml. 2-deoxy D glucose ≥99% pure was purchased from Sigma Aldrich, USA. The GOD-POD assay kit (Autospan liquid gold glucose kit) for measuring invertase activity was obtained from SPAN diagnostics (India). The primers were obtained from Chromous Biotech, Bangalore, India. The *ura4-*D18 *h^−^* strain and the haploid deletion mutant library, along with the parent control strain *ade6*-M210 *ura4*-D18 *leu1*-32 *h^+^*, were from the collection of the Bähler laboratory. It corresponds to the latest haploid disruption library version from Bioneer (v5.0), covering 98% of all non-essential genes. The generation of this mutant library has been described, and correct genotypes of the deletion mutants have been tested by PCR as described (Kim *et al.*^2010^; Rallis *et al.*^2014^). Table 1 lists the strains used in this study.

**Table 1.** List of strains used in this study.

Strain name	Genotype	Plasmid
wt	*972 h^−^*	
ura^−^	*ura4 D18 h^−^*	
pREP4X	*ura4D18 h^−^*	*pREP4X*
pODR1	*ura4 D18 h^−^*	*pODR1*
pYSP2	*ura4D18 h^−^*	*pYSP2*
Parent	*ade6 M210 ura4 D18 leu1 h+*	
*odr1*Δ	*ade6 M210 ura4 D18 leu1 SPBC215.10::KanMX h^+^*	
*^a^clr5Δ*	*ade6 M210 ura4 D18 leu1 clr5::KanMX h^+^*	
*snf5Δ*	*ade6 M210 ura4 D18 leu1snf5::KanMX h^+^*	
*fyv7Δ*	*ade6 M210 ura4 D18 leu1fyv7::KanMX h^+^*	
*pho7Δ*	*ade6 M210 ura4 D18 leu1pho7::KanMX h^+^*	
*ypa1Δ*	*ade6 M210 ura4 D18 leu1ypa1::KanMX h^+^*	

a This deletion could not be verified by PCR.

### Growth experiments

**Figure 1.**
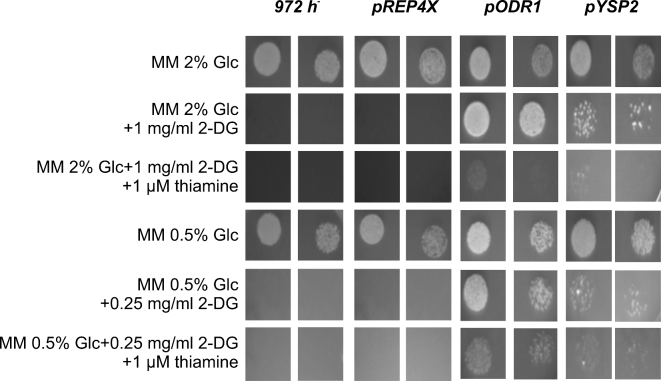
Growth of *odr1* and *ysp2* overexpressor strains on solid media in presence and absence of thiamine. Cells containing the plasmids *pODR1* and *pYSP2* under the control of the *nmt1* promoter, along with the control cells containing the plasmids *pREP4X* and wild-type cells *972*, were spotted at two different cell densities on solid MM containing 2% and 0.5% glucose (Glc). Plates contain thiamine and 2-DG as indicated (Materials and Methods). Growth resulting after plating ∼1.6 × 10^4^ and 0.4 × 10^4^ cells as 5 μl drops was assayed. When cells were diluted more, they were not growing at all or only very poorly on 2-DG plates, indicating that 2-DG resistance is highly dependent on initial cell density.

Resistance to 2-DG of strains on solid MM media was tested by growing cells in liquid MM to mid-log phase at 30°C, the cell density was normalized, and in a volume of 5 μl ∼1.6 × 10^4^ and 0.4 × 10^4^ cells were applied on the plates shown in Figs 1 and 3.

**Figure 2.**
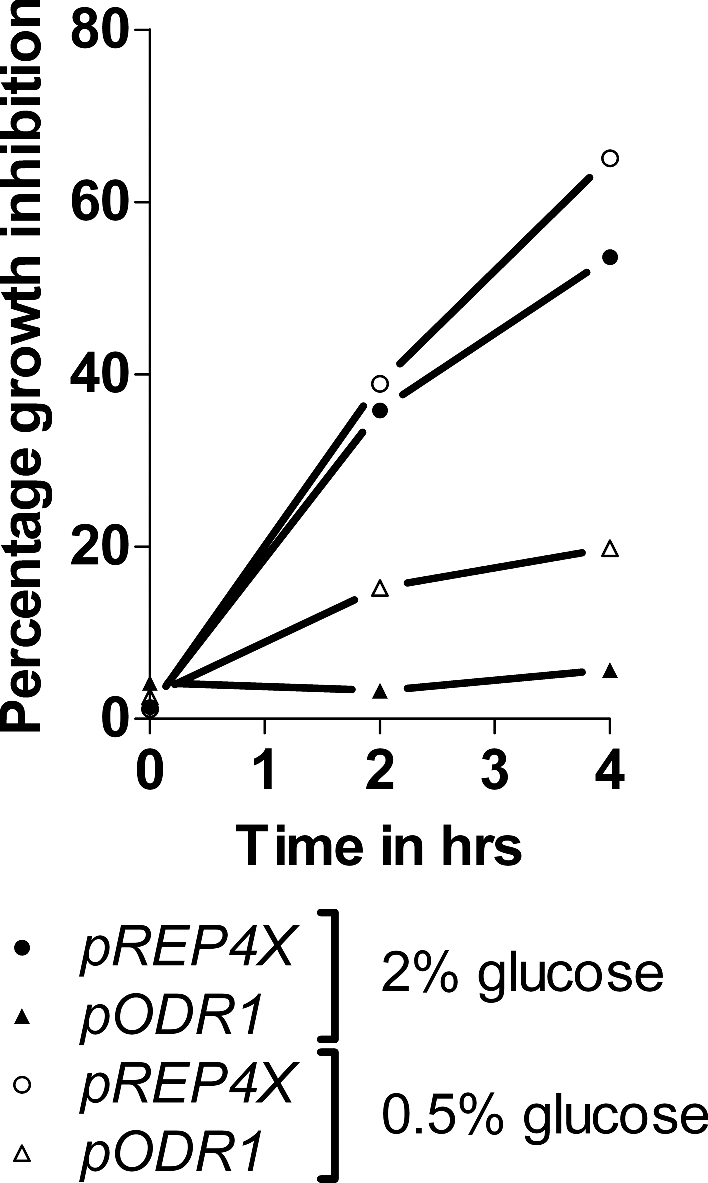
Growth inhibition by 2-DG of the *odr1* overexpressor strain in liquid medium. Cells containing the plasmids *pODR1* and control cells containing the plasmid *pREP4X* were grown at 30°C in MM containing 2% or 0.5% glucose as indicated, the presence and absence of 2-DG (0.25 mg/ml). Growth was tested at 2 and 4 h, and percentages of growth inhibition by 2-DG were plotted. The given values represent the mean of two independent experiments.

Growth in liquid media was tested by inoculating cells pre-cultured to early mid-log phase in the liquid media given in Figs 2 and 4, and growing cells at 30°C in a rotary shaker at 180–200 rpm. To estimate growth, the optical density was measured at 600 nm and percentage growth inhibition was determined by calculating the percentage decrease between growth in the absence and presence of 2-DG.

### Plasmids

To overexpress wild-type genes, we used a partial *Sau*3A genomic library ligated into the shuttle vector pURL18 as described previously (Barbet, Muriel and Carr 1992) (a gift from Viesturs Simanis). To check for accurate structure of the plasmid, we sequenced it (Chromous Biotech, India) (data not shown). Plasmid pREP4X, containing the thiamine repressible *nmt1* promoter, was obtained from Susan Forsburg (Forsburg 1993).

### Transformations

Strains were transformed by the alkali cation method as described (Moreno, Klar and Nurse 1991) or by the more rapid transformation method (Kanter-Smoler, Dahlkvist and Sunnerhagen 1994).

### Screening of the disruption strain library

Screening was performed as described (Rallis *et al.*^2014^), but with each mutant represented in quadruplicate. The initial screening was performed on EMM containing 1 mg 2-DG/ml. We re-tested the 2-DG-resistant strains on solid MM containing 1 mg 2-DG/ml. The strains examined in more detail have been tested for co-segregation of the 2-DG and kanamycin resistance marker. In addition, the deletions were validated by colony PCR as previously described (Rallis *et al.*^2014^) (Supplementary Table 2, Supporting Information; Supplementary Fig. 2, Supporting Information)

### Construction of plasmids containing odr1 and ysp2 under control of nmt1 promoter

Using standard PCR-based methods (Moreno, Klar and Nurse 1991), the genes starting with the translation start codon were fused to the *nmt1* promoter. The primers used were for *odr1* TAAGCACTCGAGATGCCGTCTAAAGAA (forward primer) and TA-AGCAGGATCCTTATTATTAATTAAAATCAGGAGGGATATTAT (reverse primer) and for *ysp1* TAAGCACTCGAGATGAAGGGT-TTAGGTCT and TAAGCAGGATCCTTATTACTAATACTTGCGAGCG. The REP4X plasmid was cut using a Xho1 and BamH1 double digest. The correct sequences of the constructs were verified by sequencing. We call the plasmids containing the *odr1 and ysp2* genes under the control of the *nmt1* promoter *pODR1* and *pYSP2*, respectively.

### Invertase assay

Cells were harvested and washed twice with ice-cold 10 mM sodium azide. Cells were pelleted and resuspended in sterile water to an optical density of 1 at 600 nm, corresponding to ∼10^7^ cells/ml. A total of 100 μl cell suspensions were aliquoted into fresh tubes, centrifuged, cell pellets were resuspended in 50 μl of 50 mM sodium acetate pH 5.1 and invertase activity was determined as described (Harkness and Arnason 2014), using the GOD-POD assay kit. Activities are given as units (micromolar glucose produced per minute) per 10^6^ cells. Statistical analysis was performed using GraphPad Prism 5^®^ (GraphPad Software Inc., La Jolla, CA, USA). Results are expressed as mean ± SEM (*n = 3*).

## RESULTS

### *odr1* and *ysp2* cause increased 2-DG resistance when overexpressed

To identify genes involved in 2-DG toxicity, we first screened for strains that are resistant to 2-DG when transformed with the wild-type gene library pURL18. Out of 77 resistant transformants, we could only isolate two intact nuclear genes. The remaining cells either contained plasmids that harbored truncated nuclear or mitochondrial genes, or the plasmids could not be recovered.

**Figure 3.**
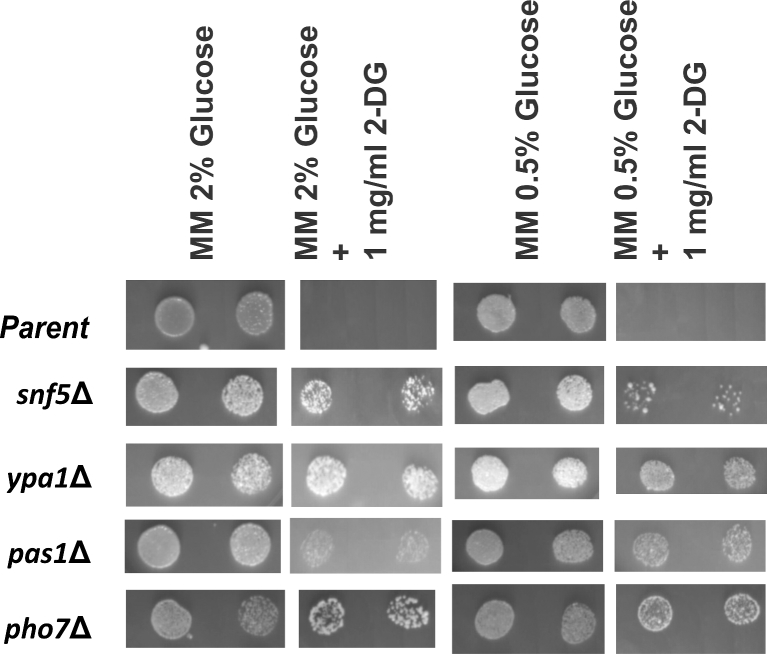
Growth of deletion mutants in absence and presence of 2-DG on solid media. The mutants as indicated, along with the parent strain (*ade6*-M210 *ura4*-D18 *leu1*-32 *h^+^*), were spotted at two different cell densities on MM and MM containing 2-DG plates, both under glucose-repressing and derepressing conditions as given for Fig. 1.

The identified genes were put under the control of the expression vector pREP4X containing the thiamine-repressible promoter *nmt1*; 2-DG resistance was then examined by growing strains in the presence and absence of thiamine. Knowing from work in *Saccharomyces cerevisiae*, growth conditions such as carbon sources can drastically alter 2-DG resistance (McCartney *et al.*^2014^). We therefore tested the strains both under glucose repressing (2%) and derepressing (0.5%) conditions, and identified two genes leading to 2-DG resistance when overexpressed. For gene SPBC215.10, called here *odr1* (for overexpression causing deoxyglucose resistance), the effect of thiamine was strong under glucose-repressing conditions, while under glucose-derepressing conditions, the overexpressing strain still weakly grew in the presence of thiamine (Fig. 1). This effect is not surprising given that full repression of the *nmt1* promoter occurs only at 15 μmolar thiamine (Forsburg 1993), a concentration that causes weak 2-DG resistance for unknown reasons (unpublished results). The other gene, *ysp2*, showed only weak 2-DG resistance when overexpressed (Fig. 1). The few resistant colonies growing on 2-DG-containing plates probably represent cells containing high plasmid copy numbers. The resistant colonies were unlikely to reflect spontaneous 2-DG-resistant mutants, given that no resistant cells were observed for the wild-type cells, parent control cells and the deletion cells (Fig. 3). Given its weak resistance, we did not further study *ysp2*.

To quantify 2-DG resistance of the *odr1* overexpressor strain, we tested its growth in liquid medium. Neither the wild-type nor parent strain did grow with 1 mg/ml 2-DG, so growth was examined at 0.25 mg/ml 2-DG. Cells were inoculated in the presence and absence of 2-DG and growth was measured after 2 and 4 h incubation. Figure 2 shows the growth inhibition by 2-DG. The data confirm that *odr1* overexpression leads to 2-DG resistance, although the effect was weaker in liquid low-glucose medium than in high-glucose medium.

Knowing that overexpression of *odr1* causes resistance to 2-DG, we tested whether the corresponding deletion mutant (*odr1*::*kanMX ade6*-M210 *ura4*-D18 *leu1*-32) was more sensitive than its parent strain. However, quantitative measurements in liquid medium showed no increased sensitivity to 2-DG for the *odr1* mutant (data not shown).

### Identification of four genes causing 2-DG resistance when deleted

In a complementary approach to identify genes involved in 2-DG toxicity, we pre-screened a haploid deletion library for mutants resistant to 2-DG on EMM plates containing 1.0 mg/ml 2-DG. This first screen identified 14 mutants exhibiting 2-DG resistance phenotypes in both of the two independent repeats carried out (Supplementary Fig. 1, Supporting Information; Supplementary Table 1, Supporting Information). We also performed a second screen for mutants resistant to 2-DG on EMM plates containing the lower dose of 0.5 mg/ml 2-DG. This screen identified 59 mutants exhibiting 2-DG resistance phenotypes in both of the two independent repeats carried out. The hits of the second screen included all 14 mutants of the first screen (Supplementary Table 1, Supporting Information).

To characterize growth of the 14 resistant mutants from the first screen, we tested them under the same physiological conditions as the *odr1* overexpressor strain in MM containing 2-DG (1.0 mg/ml). For seven mutant strains, deleted for the genes *plc1, iec1, adn3, vps38, rxt3, trk2 and moe1*, resistance to 2-DG was weak (data not shown), while one mutant, deleted for the gene *snz1*, did not grow after re-streaking on either MM or EMM for unknown reasons (data not shown). These differences in resistance and growth under the two conditions tested highlight the effect that physiological conditions can have for experiments involving glucose metabolism. For one mutant, deleted for the gene *fyv7*, the kanamycin resistance did not co-segregate with 2-DG resistance, revealing that the resistance was not caused by the deletion.

**Figure 4.**
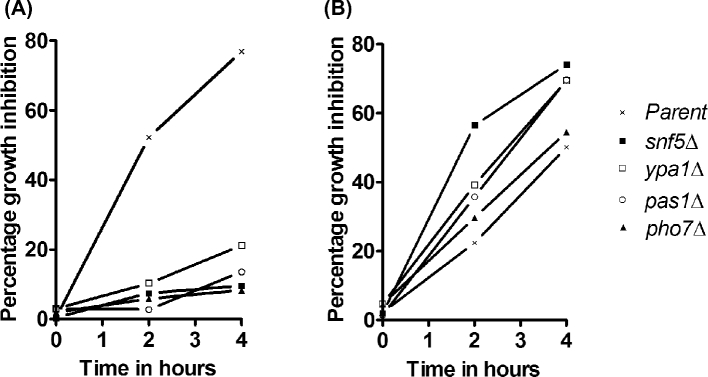
Growth inhibition of deletion mutants by 2-DG in liquid medium. The mutants as indicated, along with the parent strain (*ade6*-M210 *ura4*-D18 *leu1*-32 *h^+^*), were grown at 30°C in MM medium containing 2% glucose (**A**) or 0.5% glucose (**B**) in the presence and absence of 2-DG (0.25 mg/ml). Growth was tested after 2 and 4 h and percentages of growth inhibition by 2-DG were plotted. The data represent the mean of two independent experiments.

Four of these 14 strains identified in the screen exhibited moderate to strong resistance under glucose-repressing conditions on MM plates containing 1.0 mg/ml 2-DG. A fifth strain, *clr5Δ*, also caused 2-DG resistance, but we could not confirm the deletion by PCR genotyping (Supplementary Fig. 2, Supporting Information). The four PCR-verified strains were deleted for the following genes: *snf5, ypa1, pas1* and *pho7*. For all of these mutants, the kanamycin marker co-segregated with the 2-DG resistance phenotype (data not shown) shows that the resistance is caused by the gene deletion. Examination of cell morphologies of the four resistant mutants did not reveal any evident differences to wild-type cells (data not shown). Except for the mutant *snf5*Δ, which has previously been reported to be 2-DG resistant (Monahan *et al.*^2008^), the other four mutants also grew well under derepressing conditions on solid medium (Fig. 3). Growth tests of the four mutants in liquid medium exhibited strong 2-DG resistance under glucose-repressing conditions (Fig. 4A). On the other hand, these mutants did not show any 2-DG resistance under derepressing conditions, with most showing even higher sensitivity than the parental strain (Fig. 4B). We assume that osmotic stabilization effects acting in colonies but not in liquid medium may be responsible for the different growth behavior.

### 2-DG-resistant mutants exhibit altered invertase expression

**Figure 5.**
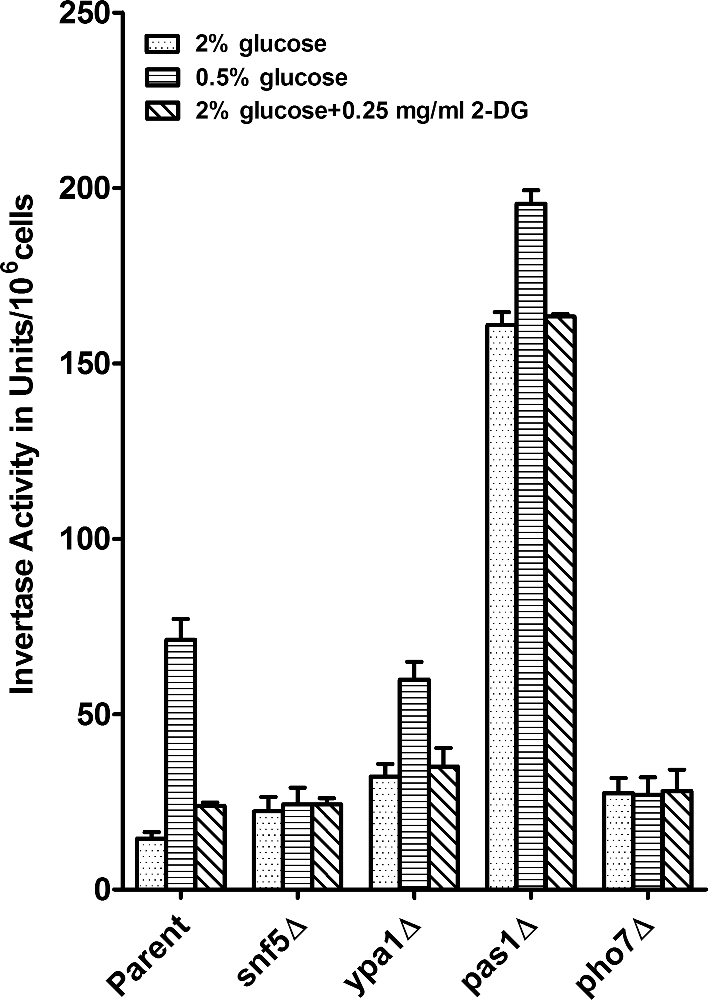
Invertase activity of the 2-DG-resistant mutants. Cells from the 2-DG-resistant deletion mutants and the corresponding parent strains *ade6*-M210 *ura4*-D18 *leu1*-32 *h^+^* were grown in MM medium containing 2% or 0.5% glucose and in 2% glucose medium containing 2-DG as indicated. Invertase activity was determined and is given as activity per cells (Materials and Methods). The values given represent the mean of three measurements, with samples from each measured in five replicates, originating from three independent experiments. Error bars represent SEM (*n = 3*).

The invertase Inv1 of *Schizosaccharomyces pombe* is a glucose-repressible cell wall glycoprotein (Moreno *et al.*^1985^). Inv1 expression is partially diagnostic for the control of glucose metabolism and particularly for the state of glucose repression. We tested the Inv1 activity in the 2-DG-resistant mutants grown under repressed and derepressed conditions. The mutant strains varied in their activity levels, both in the repressed and derepressed state, when compared to the parent strain, with the greatest upregulation observed in *pas1*Δ. The deletion mutants *snf5*Δ and *pho7*Δ were more affected in glucose derepression, whereas *ypa1*Δ was more affected under glucose repression (Fig. 5). In summary, comparing the ratios of the activities of cells grown under high- and low-glucose conditions, the mutants and the *odr1-*overexpressing strain featured a more constitutive invertase phenotype than the control strains (Table 2). Examination of the corresponding *odr1*Δ mutant revealed a hyperderepression (Table 2). These results indicate that the *odr1* gene, in its wild-type configuration, plays a role in glucose repression.

**Table 2.** Ratios of invertase activity of 2-DG-resistant mutants under glucose-repressed and derepressed conditions and in the presence of 2-DG.

Strain	Fold increase of invertase activity from 2% to 0.5% glucose	Fold increase of invertase activity in 2% glucose containing 2-DG
*pREP4X*	3.9 ± 0.26	2.2 ± 0.19
*pODR1*	1.6 ± 0.06	1.3 ± 0.07
*Parent*	5.0 ± 0.44	1.7 ± 0.6
*odr1*Δ	6.9 ± 0.83	3.6 ± 0.61
*snf5*Δ	1.1 ± 0.01	1.2 ± 0.19
*ypa1*Δ	1.9 ± 0.22	1.1 ± 0.05
*pas1*Δ	1.2 ± 0.004	1.0 ± 0.03
*pho7*Δ	1.0 ± 0.08	1.0 ± 0.06

pREP4X: control strain for overexpressor strain *pODR1.*

Parent: parent strain of deletion strains (*ade6*-M210 *ura4*-D18 *leu1*-32 *h^+^*).

Different ratios of activities in *pREP4X* and parent strains are due to plasmid effects. Activity was measured in MM containing 2%, 0.5% or 2% glucose plus 0.25 mg/ml 2-DG (see Materials and Methods) and the ratios ±SEM (*n = 3*) are indicated here.

We also tested the effect of 2-DG on the regulation of invertase activity under glucose-repressed conditions. In the parent strains, 2-DG enhanced Inv1 activity, in contrast to the situation in *S. cerevisiae* where 2-DG represses its activity (Randez-Gil, Prieto and Sanz 1995). Similar results were obtained for all tested strains, including control strains 972, *ura4*-D18 and the pREP4X-containing strain (data not shown). These results suggest that 2-DG is mimicking glucose starvation. For the 2-DG-resistant mutants, the effects of 2-DG on Inv1 expression are qualitatively similar as for low glucose (Table 2).

## DISCUSSION

The genetics of 2-DG resistance is complicated as 2-DG and its metabolic products are involved in a variety of processes. This makes it difficult to understand mechanism of 2-DG actions or its resistance. We identified the *Schizosaccharomyces pombe odr1* gene causing strong 2-DG resistance when overexpressed. Despite examining many 2-DG resistant transformants, we could detect only one gene exhibiting this strong overexpression phenotype. In *Saccharomyces cerevisiae*, a similar screening yielded two genes, *DOG1* and *DOG2* (Randez-Gil *et al.*^1995^). These findings suggest that the number of genes whose overexpression confers 2-DG resistance is limited. On the other hand, we uncovered several deletion mutants that render cells resistant to 2-DG. Among 14 initially selected mutants, four mutants conferred moderate to good resistance to 2-DG. By screening at a 2-fold lower 2-DG concentration, we isolated 59 resistant mutants (Supplementary Fig. 1, Supporting Information; Supplementary Table 1, Supporting Information). These findings suggest that control of 2-DG resistance is a complex process involving many genes, considering also that we have not tested any genes that are essential for growth. In a similar screen performed in *S. cerevisiae*, 19 deletion mutants that are resistant to 2-DG have been reported (Ralser *et al.*^2008^), although it has later been suggested that 16 of these mutants are not actually causing 2-DG resistance (McCartney *et al.*^2014^). Out of the three remaining genes, deletion of *HXK2* and *REG1* provides strong resistance on glucose-rich media, whereas deletion of *LSM6* is only weakly resistant (Neigeborn and Carlson 1987; Ralser *et al.*^2008^; McCartney *et al.*^2014^).

The transcription of the invertase gene is repressed by the presence of glucose (Tanaka *et al.*^1998^). The deletion mutants are affected to different degrees in the control of invertase activity (Fig. 5). According to the invertase activity ratios in the repressed versus derepressed states, the mutants exhibit constitutive invertase activity (Table 2). The corresponding proteins could be directly or indirectly involved in this control. The invertase activity data also indicate that 2-DG induces a glucose starvation signal and thus derepresses the invertase activity, while the deletion mutants resistant to 2-DG show more constitutive invertase activities across different conditions (Fig. 5).

Decreased growth on glucose has been reported for mutants deleted for *clr5, ypa1* and *pho7*, consistent with these genes being defective in the control of glucose metabolism (Vachon *et al.*^2013^; Doi *et al.*^2015^). The genes *snf5, pas1 and pho7* are involved in transcription (Table 3), raising the possibility that mutants of these genes prevent some changes in gene expression induced by 2-DG that could promote resistance to 2-DG. The *snf5, ypa1* and *pas1* genes show orthologs in *S. cerevisiae*, but the corresponding mutants have not been reported to be 2-DG resistant. The *S. cerevisiae* protein Snf1 is involved in controlling 2-DG sensitivity (O'Donnell *et al.*^2015^), but its *S. pombe* ortholog, Ssp2, has neither been reported to be involved in resistance to 2-DG, nor did it come up in our screen.

**Table 3.** List of genes and their annotations identified in this study**.**

Gene standard name	Gene product	Molecular function	References
*snf5*	Chromatin remodeling (SWItch/Sucrose Non-Fermentable; SWI/SNF) complex subunit	Regulation of transcription and glucose import	Monahan *et al.* (2008)
*ypa1*	Protein phosphatase type 2A regulator	Regulation of protein phosphatase activity	Goyal and Simanis (2012)
*pho7*	Transcription factor	Regulation of RNA polymerase II-mediated transcription	Estill, Kerwin-Iosue and Wykoff (2015)
*pas1*	Cyclin	Regulation of G1/S transition of cell cycle	van Slegtenhorst, Mustafa and Henske (2005)

Only the most important annotations of the genes are given. For more details refer to *S. pombe* genome data and annotations database (Wood *et al.*^2012^).

Even though some molecular functions of the corresponding proteins have been described (Table 3), we do not have a straightforward explanation for how the genes identified affect 2-DG resistance and glucose repression when deleted. The protein Snf5 is a subunit of the SWI/SNF chromatin remodeling complex involved in transcription and chromatin-related processes. Interestingly, the human Snf5 ortholog is involved in cancer development (Roberts and Orkin 2004). Deletion of *snf5* leads to increased mRNAs encoding hexose transports (Monahan *et al.*^2008^), which could explain the increased 2-DG resistance in the high-glucose condition only, where increased influx of glucose may titrate out 2-DG (Figs 3 and 4). This result is of interest in light of findings in *S. cerevisiae* that 2-DG acts by reducing the levels of hexose transporters by α-arrestin-mediated endocytosis and degradation in vacuoles (O'Donnell *et al.*^2015^). Deletion of *snf5* in *S. pombe* may thus counteract this effect of 2-DG. Except for the *snf5* deletion, the other four deletion mutants showed resistance to 2-DG in both high- and low-glucose conditions, suggesting that these gene deletions affect resistance by a different mechanism.

We speculate that the *S. pombe* Odr1 hydrolase might function in detoxifying toxic forms of 2-DG, similar to the *S. cerevisiae* Dog1/Dog2 proteins that encode haloacid dehalogenase-like (HAD-like) hydrolase enzymes exhibiting specific 2-DG-6-phosphatase activities and thus render cells 2-DG resistant when overexpressed. The physiological function of these enzymes is not known (Sanz, Randez-Gil and Prieto 1994; Randez-Gil, Prieto and Sanz 1995; Randez-Gil *et al.*^1995^). An HAD-like hydrolase domain is also present in Odr1, besides a Cof-subfamily domain believed to be required for enzymes acting on phosphorylated sugars (Marchler-Bauer *et al.*^2015^). It is therefore plausible that Odr1 detoxifies the toxic form of 2-DG. Notably, these proteins have opposite effects on invertase expression: overexpression of Odr1 causes derepression of *S. pombe* invertase, whereas overexpression of Dog1 causes repression of *S. cerevisiae* invertase (Randez-Gil, Prieto and Sanz 1995).

A BLAST search (Altschul *et al.*^1990^) for Dog1/Dog2 orthologs in *S. pombe* yielded protein SPCC1020.07 (annotated as a pseudouridine 5΄ phosphatase), and a search for Odr1 paralogs in *S. pombe* revealed a protein (SPAC25B8.12c) with 45% identity. None of these proteins, however, did come up in our screen for genes causing 2-DG resistance during overexpression, but the deletion mutant of SPCC1020.07 renders the cells weakly 2-DG resistant (Supplementary data, screening at 0.5 mg/ml 2-DG, Supporting Information).

The *S. pombe* orthologs of genes conferring resistance when overexpressed or deleted in *S. cerevisiae* (*DOG1/DOG2, REG1* and *HXK2*) did not come up in our screen. Moreover, the *odr1* gene, rendering *S. pombe* cells 2-DG resistant, shows the opposite effect on invertase activity to the *DOG1* gene, rendering *S. cerevisiae* 2-DG resistant. Notably, the finding that 2-DG derepresses the invertase activity in *S. pombe*, unlike in *S. cerevisiae* where it is repressed (Randez-Gil, Prieto and Sanz 1995), suggests that control of 2-DG resistance in the two yeasts is achieved by at least partially different mechanisms. The mechanisms by which the identified genes affect 2-DG-induced gene expression, sensitivity and glucose repression in *S. pombe* require further studies.

## Supplementary Material

Supplemental material Supplementary data are available at *FEMSYR* online.
