# Supplementary figures and images for: Identification of nuclear genes affecting 2-Deoxyglucose resistance in Schizosaccharomyces pombe
Source: FEMS Yeast Res. 2016 Jul 31;16(6):fow061. doi: 10.1093/femsyr/fow061 (PMC5452730; doi:10.1093/femsyr/fow061)

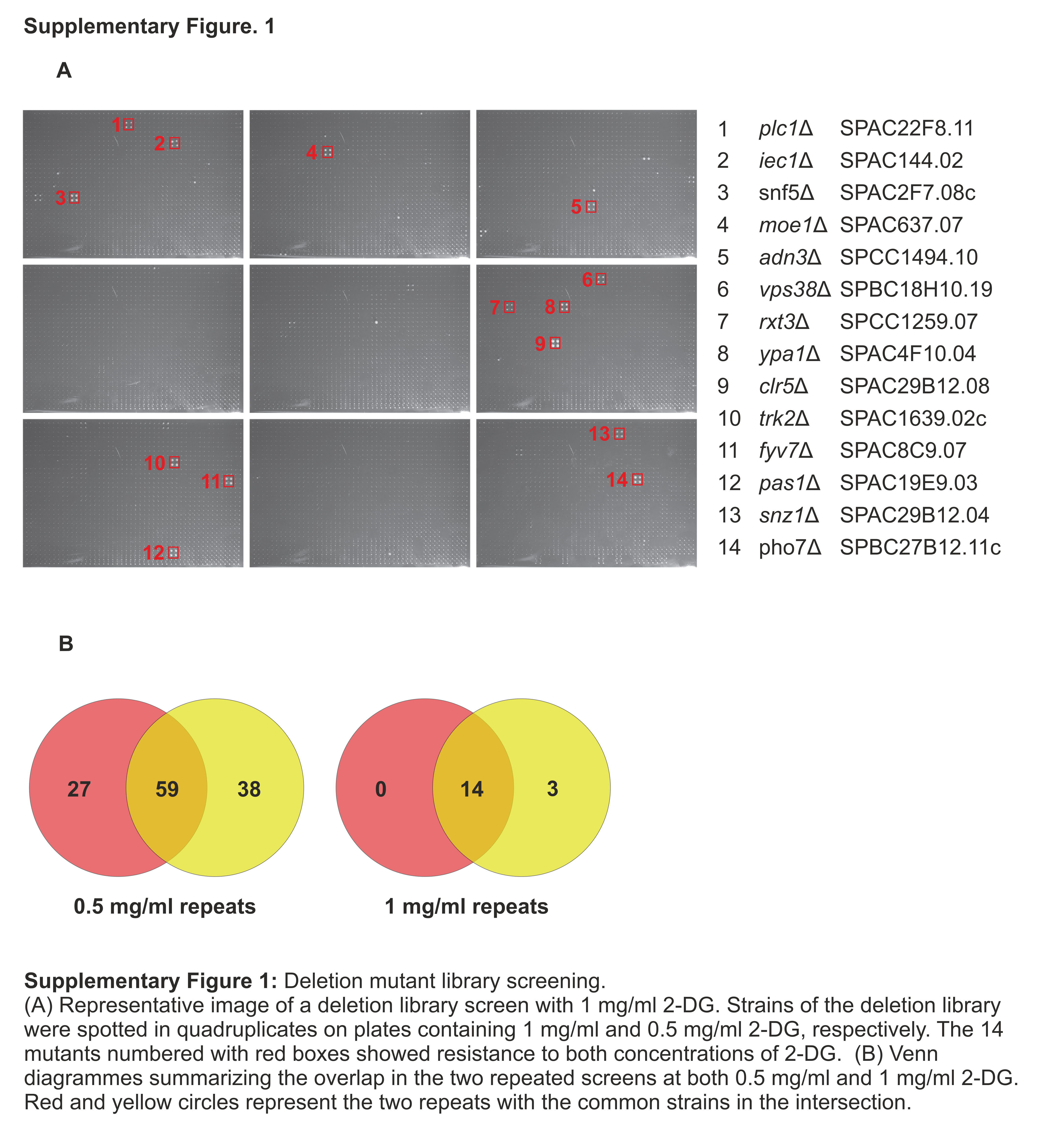

Supplement: Supplemental material — Supplementary data are available at FEMSYR online. [file fow061_supp.zip › Supplementary_Figure_1.tif]

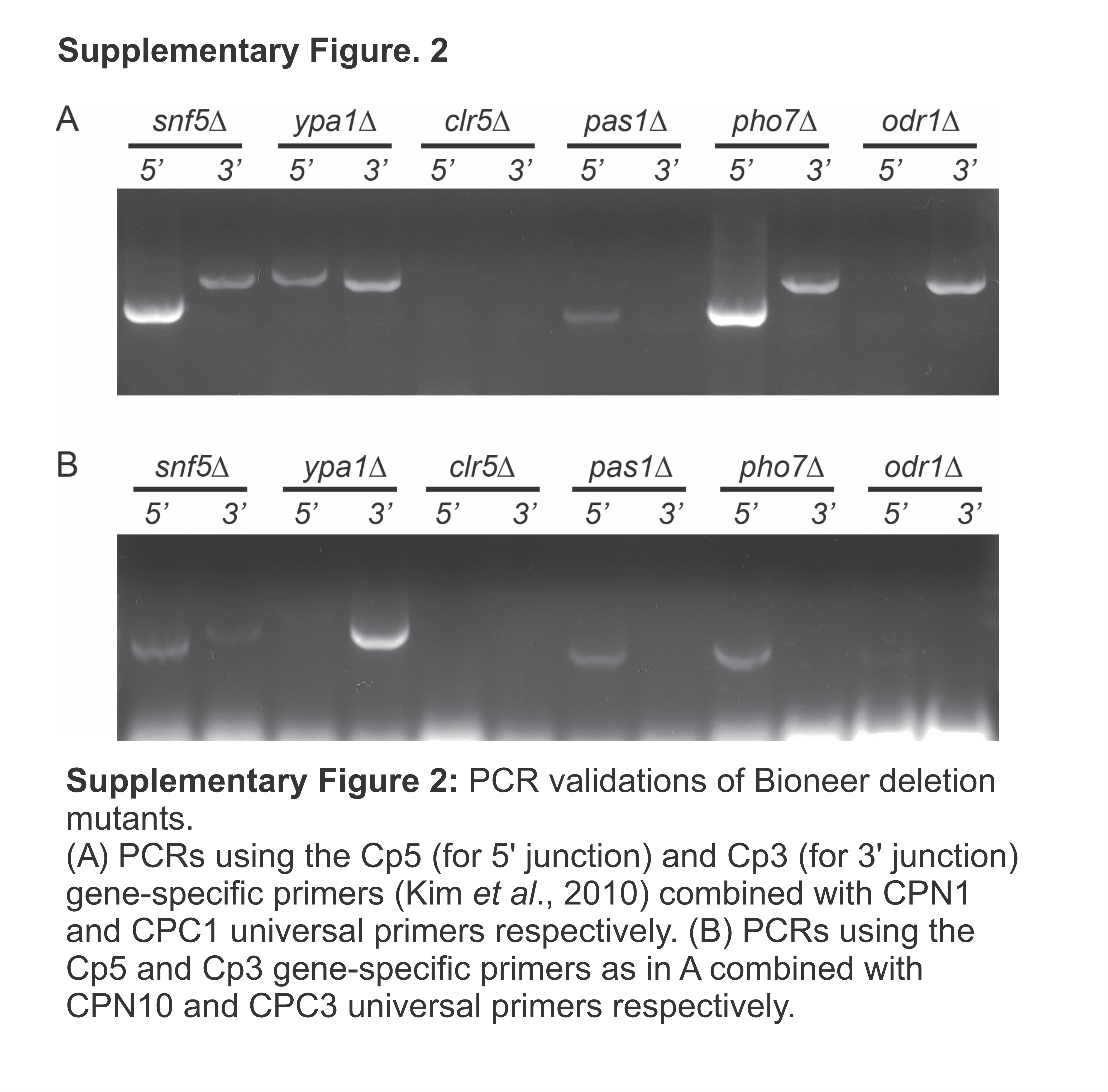

Supplement: Supplemental material — Supplementary data are available at FEMSYR online. [file fow061_supp.zip › Supplementary_Figure_2.tif]
