# Supplementary material for: Identification of nuclear genes affecting 2-Deoxyglucose resistance in Schizosaccharomyces pombe
Source: FEMS Yeast Res. 2016 Jul 31;16(6):fow061. doi: 10.1093/femsyr/fow061 (PMC5452730; doi:10.1093/femsyr/fow061)
Supplement: Supplemental material — Supplementary data are available at FEMSYR online. [file fow061_supp.zip › Supplementary_Table_2.doc]

**Supplementary Table 2:** Primers used for validation of deletion mutants.

| Gene specific primers | | | |
| --- | --- | --- | --- |
| Gene | Cp5 | | Cp3 |
| *odr1* | CCTTCTCACTACAAACGCATACCCT | | CCAAATTGGATGAGGAGGGTAAAAG |
| *snf5* | TCCTTTAGTAACTCACTCTCCGTCG | | TATGATTCCATGGTTTTGCATCCAT |
| *clr5* | GCGTTGAAGTTGTCTTTCGCTACTT | | AGGATCTCGTATACGAAAGTACCGG |
| *ypa1* | TGTTTTTCAGTTTGGTAAAAACTGG | | ATTGCAGCAATTGGTATTCATTTAC |
| *pho7* | TTATACTGCTTTGACGAGATTGAGC | | ACTTCTTTCGTCATCTGTACGATCA |
| *pas1* | GCTTGAAGATTCGATTTCAAGCAAT | | CAACATTAGGATCGGTGGAATCATA |
| Universal primers | | | |
| CPN1 | | CGTCTGTGAGGGGAGCGTTT | |
| CPN10 | | GATGTGAGAACTGTATCCTAGCAAG | |
| CPC1 | | TGATTTTGATGACGAGCGTAAT | |
| CPC3 | | GGCTGGCCTGTTGAACAAGTCTGGA | |
